# Uncommon Canal Anatomy: Identification and Treatment of Middle Distal Canal in Mandibular First Molars—A Report on Two Cases

**DOI:** 10.1155/crid/9052971

**Published:** 2026-09-08

**Authors:** Hend Mahmoud Abou El Nasr

**Affiliations:** ^1^ Endodontics, Oral Health Department, Dubai Health, Dubai, UAE; ^2^ Endodontics, Faculty of Dentistry, Cairo University, Giza, Egypt, cu.edu.eg

**Keywords:** anatomical variations, Emirati population, mandibular first molars, middle distal canals, root canal configuration

## Abstract

Mandibular molars often exhibit high variation in canal anatomy. Failing to identify these variations can negatively impact treatment outcomes. This report describes two cases of mandibular first molars with middle distal canals. In Case 1, the patient was a United Arab Emirates (UAE) national who presented with symptomatic irreversible pulpitis; whereas in Case 2, the patient had a previously treated tooth and chronic apical abscess. Both cases were treated with nonsurgical root canal therapy, and the extra canals were identified using magnification and troughing with ultrasonic tips. In Case 2, healing was observed at the 6 months and 1 year follow‐up. This report highlights the lack of data on the incidence of middle distal canals in Emirati population and underscores the importance of meticulous canal exploration and the use of enhanced diagnostic aids to detect atypical canal anatomy.

## 1. Introduction

Missed anatomy is a potential reason for failure of root canal treatment (1–5). This incident is often the result of either insufficient knowledge of root canal anatomy or failure to locate and detect additional canals. The risk of missed anatomy is even higher in mandibular first and second molars due to the well‐documented complexity of their canal systems (6). In fact, the incidence of untreated canals in these teeth was reported to be around 9.5%–23% (2–4, 7), and the most frequently unidentified anatomy in mandibular molars is middle mesial canals (8).

The prevalence of middle canals in various populations was investigated in a systematic review by Bansal et al. (9) who found that the presence of middle mesial canals ranged from 0.26% to 53.8%, whereas that of middle distal canals was 0.0%–10%.

Clearing (10), scanning electron microscope (11), periapical radiographs (12), and computed tomography (CT) (11) were the most used tools for dissecting root canal anatomy. However, the introduction of 3D imaging allowed on site clinical inspection of canal morphology and configurations. Tredoux et al. (13) found middle mesial canals in 20% and middle distal canals in 7% of CBCT scans of a South African subpopulation. Whereas only one middle distal canal (0.7%) was observed in 145 CBCT scans of mandibular first and second molars of a Saudi subpopulation (14). Microcomputed tomography of extracted mandibular first molars revealed middle mesial canals in 51% and middle distal canals in 14.6% of an Egyptian subpopulation (15).

Moreover, although the middle mesial canal was largely investigated, few cases reported the presence of a third canal in the distal root of mandibular molars. In 1985, Martinez‐Berna and Badanelli (16) found middle canals in both the mesial and distal roots. Kottoor et al. (17) resorted to the dental operating microscope to locate a middle distal canal in a mandibular first molar that looked normal on periapical radiograph; later, Jabali described a mandibular first molar with middle mesial and middle distal canals after selective troughing under high magnification (18). Whereas Venumuddala and colleagues confirmed the presence of an additional distal canal in the distal root of a mandibular first molar with the aid of sliced computed tomography (19).

Failure to clinically identify these variations is probably because the diameter and volume of middle canals are usually much smaller than those of the main canals (20). Moreover, the entrance to middle canals is often difficult to find, because they are mostly located at the cementoenamel junction or 1–2 mm deeper (8, 21).

These anatomical characteristics of middle canals complicate canal detection, negotiation, instrumentation, and disinfection, which increases the likelihood of residual microbial contamination and eventually compromises treatment outcome.

To date, no reports were documented from the United Arab Emirates describing such variant in the root canal system. Hence, the purpose of this article is to present two cases of different ethnicities for permanent mandibular first molars, each with two roots. The mesial root presents with two root canals, whereas the distal roots exhibited 3 root canals.

## 2. Case Reports

This case report was prepared in accordance with CARE guidelines (22). Ethical approval for this report was not required, as it describes only two individual anonymized cases.

### 2.1. Case 1

**Figure 1:**
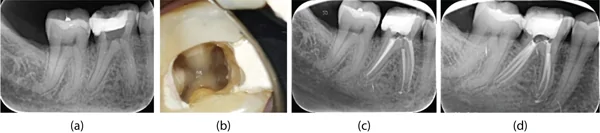
(a) Preoperative radiograph of a mandibular first molar. (b) Photograph of the access cavity showing orifices of three canals in the distal root. (c) Postoperative radiograph with a straight view. (d) Shifted view displaying three independent canals in the distal root.

A 40‐year‐old male patient (United Arab Emirates national) with no significant medical history was referred from general dental practitioner for root canal treatment of a mandibular right first molar. The tooth was diagnosed with symptomatic irreversible pulpitis (severe pulpitis) and normal periapical tissues based on patient′s chief complaint of severe pain following the placement of composite restoration, as well as the results obtained from clinical and radiographic examinations. Cold testing elicited exaggerated prolonged response, whereas clinical examination of periapical tissues revealed normal response to palpation, slight tenderness to percussion, and normal probing depths, as well as absence of any mucosal or gingival abnormalities. Preoperative periapical radiograph showed a previously opened access cavity with widening of the periodontal ligament space around the distal root (Figure 1a). A written informed consent was obtained from the patient after explaining the case and describing the procedure.

The general dental practitioner performed pulpectomy as an emergency procedure and affirmed the presence of four canals but also reported blocked distal canals.

Following administration of local anesthesia with 2% lidocaine hydrochloride with 1:80.000 adrenaline (Lignospan Special, Septodont, France) and rubber dam isolation, the pulp chamber was inspected with a DG‐16 explorer under a surgical operating microscope (Leica Microsystems M320, GmbH, Germany). Moreover, ultrasonic tip (Start X, Dentsply Maillefer, Ballaigues, Switzerland) was used for selective removal of dentin between the previously located distobuccal and distolingual canals, which revealed an additional canal between them (Figure 1b). Working lengths in all five canals were determined with the aid of electronic apex locator (Root ZX II, United States), and instrumentation was performed using ProTaper Next (Maillefer, Dentsply, Switzerland) to an apical size 30/0.07 (×3) with copious irrigation of 2% sodium hypochlorite (NaOCl). The canals were dried with paper points and obturated with laterally condensed gutta percha (Maillefer, Dentsply, Ballaigues, Switzerland) and Sealapex (Kerr Dental, Orange, California, United States) root canal sealer (Figure 1c,d). A temporary filling material (IRM, Dentsply Sirona, United States) was mixed and placed to seal the access cavity until a permanent restoration was placed.

### 2.2. Case 2

**Figure 2:**
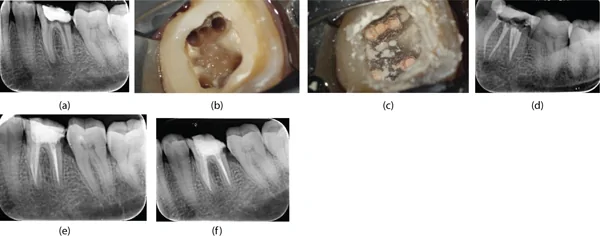
(a) Preoperative radiograph of a mandibular first molar showing a periapical radiolucency related to the mesial root. (b) Access cavity displaying three canals in the distal root. (c) Obturation of the three canals as seen under dental operating microscope. (d) Postoperative radiograph showing confluent canals in the distal root. (e) 6 months follow‐up showing resolution of the periapical radiolucency. (f) 1 year follow‐up showing reestablishment of normal bone trabeculation.

A 54‐year‐old male patient (Indian national) with noncontributory medical history presented with intraoral buccal swelling related to his mandibular left first molar. The patient was asymptomatic, but clinical examination revealed a mandibular left first molar sealed with a temporary filling, a small localized buccal swelling with slight tenderness to percussion and palpation, and probing depth of about 4 mm on the distal side, yet the tooth exhibited mobility within normal limits. Radiographic examination showed previous root canal obturation deemed unacceptable due to the short filling in the mesial canals and the evident presence of voids, in addition to a well‐defined radiolucency related to the mesial root and horizontal bone loss on the distal side (Figure 2a). Accordingly, the diagnosis was confirmed as previously obturated tooth with chronic apical abscess.

After obtaining a written consent from the patient, an inferior alveolar nerve block injection of 2% lidocaine hydrochloride with 1:80.000 adrenaline (Lignospan Special, Septodont, France) was administered followed by isolation of the tooth with rubber dam. The temporary filling was removed, and the orifices of four canals were located in the floor of the pulp chamber. Old root canal filling was removed from all canals using Protaper Universal Retreatment files (Dentsply Sirona, United States) and copious irrigation of 2% NaOCl. The floor of the pulp chamber was then magnified with Leica surgical operating microscope and inspected using DG‐16 explorer. This revealed an additional canal midway between distobuccal and distolingual canals (Figure 2b). Patency was obtained and working length was determined using Root ZX II electronic apex locator. The five root canals were instrumented using Protaper Next to size ×3 (30/07) and irrigation with 2% NaOCl after each file. A calcium hydroxide (Calcipast, Cerkamed, Poland) intracanal dressing was placed in all canals before building the tooth with glass ionomer filling material (Photac Fil Quick Aplicap, 3M GmbH, Germany).

The patient was seen again after a week where he confirmed absence of symptoms and his ability to chew normally on this tooth. Clinical inspection demonstrated the absence of the buccal swelling and a normal appearance of the buccal mucosa. Therefore, the canals were obturated using lateral condensation of gutta percha with Sealapex root canal sealer (Figure 2c,d) (Kerr Dental, Orange, California, United States). The cavity was then sealed with intermediate restorative material (IRM, Dentsply Sirona, United States), and the patient was referred to the prosthodontist for planning a full coverage restoration.

The patient was called for follow‐up after 6 months, during which he expressed satisfaction with absence of pain and the normal function of the tooth. The clinical picture did not change from the previous visit except for the presence of a core sealing the clinical crown, whereas radiographic examination revealed reduction in the size of the periapical radiolucency around the mesial root, and fiber posts placed in mesiobuccal and distobuccal canals (Figure 2e). The patient was seen again after 1 year, where the periapical radiograph displayed complete healing of the periapical tissues (Figure 2f).

## 3. Discussion

A recent meta‐analysis concluded that untreated missed canals significantly increased the likelihood of posttreatment apical periodontitis in root filled teeth, highlighting the importance for thorough canal detection and treatment (5).

The present article demonstrates two cases of mandibular first molars that exhibited additional anatomy of rare occurrence. Case 1 presented as completely asymptomatic; nonetheless, an additional canal was located in the groove between the distobuccal and distolingual canals upon examination of the pulp chamber under the surgical operating microscope. Routine steps of instrumentation and obturation were undertaken, and the final radiograph showed a distal root with three separate exits. This configuration corresponds to Type VIII in Vertucci classification, where three distinct canals run from pulp chamber to the canal terminus (3–3 configuration) (23). According to Pomeranz et al. (9, 24), it is described as an independent canal that originated as a separate orifice and runs independently from the orifice to the apex. This tooth belonged to an UAE citizen, where no current information exists regarding the incidence, prevalence, or configuration of this variation within the population.

Conversely, middle distal canals were reported in Europeans, Asians, Africans (13), and South and North American cohorts (9). However, the Indian population showed the highest pooled prevalence of 5.70% (25). Goel et al. (26) reported a 1.7% incidence in the mandibular first molar using a radiographic method, whereas Neelakantan et al. (27) found a 0.57% incidence in mandibular second molars using a tooth clearing method. Furthermore, most of the cases reported in the literature came from the Indian subcontinent (17, 19, 28–31) and were reported in mandibular first molars.

Case 2 was a 54‐year‐old Indian native who presented with an intraoral localized swelling and a periapical lesion related to the mesial root. On inspection of the floor of the pulp chamber, it seemed more logical to find the extra anatomy related to the mesial root, but to the author′s surprise, it is the middle distal canal that revealed itself. In fact, healing of the periapical tissues occurred over a year, as evidenced by restoration of normal bone trabeculation.

In this case, the distal root had three distinct root canal orifices with single apical termination, where the middle distal canal joined the mesiobuccal and mesiolingual canals in a confluent form before exiting from the apex (24) and could be described as Type XVIII canal configuration according to Sert and Bayirli (32). This aligns with previous findings confirming that confluent configuration was more prevalent among different middle canal presentations (15, 25) and corroborated previous reports (17, 30).

Both cases reported in this article are mandibular first molars, aligning with previous studies that concluded higher prevalence of middle canals in mandibular first molars than second molars (15, 25). However, one case was for an Indian male patient belonging to the highest reported prevalence group among studied ethnicities. In contrast, the other case was for an Emirati citizen, where no current information exists regarding the incidence or prevalence of such type of anatomical variation. Only reports (25) from the Arab and gulf region were documented showing very low prevalence of middle distal canals in Jordanian (0.9%) (25) and Saudi populations (0.7%) (14).

In both cases, the orifice of the extra canal was located after troughing under high magnification, reinforcing previous recommendations calling for the imperative use of the surgical microscope for the detection of extra canals (6, 25), as well as the benefit of ultrasonic tips in selective removal of dentin (33). However, it is worth noting that ultrasonic tips should be used with caution and at adequate power, because aggressive and indiscriminate cutting may lead to perforation (34).

Further, an increased number of canals within a root is typically accompanied by smaller canal diameters and more complex configuration, which presents significant challenge during negotiation and biomechanical preparation (20, 35). All canals in both cases were instrumented with the same rotary system (PTN), because it proved to be one of the contemporary file systems preserving canal anatomy after instrumentation (36), in addition to allowing efficient disinfection (37). The combination of flexibility gained by its M‐Wire alloy, off‐centered rectangular cross‐section, as well as swaggering movement reduced contact with canal walls, thereby minimizing excessive dentin removal and preserving original canal anatomy (38).

This case report highlights an infrequent variant of canal anatomy that is not fully addressed in the literature. It demonstrated a practical approach for managing the reported cases with the integration of magnifying aids and prudent use of ultrasonic tips and documents favorable outcomes following the location and management of these canals. This report also discloses the lack of information about the true prevalence of this variation among Emirati population.

However, the main limitation of this report is the limited number of cases, which limits the generalizability of the findings. Nevertheless, detailed documentation of the cases may serve as the basis for further investigation, which clarifies the true prevalence of this variation, whereas on the other side, clinicians are encouraged to use appropriate tools to detect additional anatomy.

## 4. Conclusion

Due to the inherent nature of case reports, little evidence is provided regarding the incidence of middle distal canals in Emirati population, hence the need for cross‐sectional studies filling this gap in the literature. Moreover, this case report highlights the importance of the use of surgical operating microscope which substantially improves detection of hidden canal anatomy.

## Author Contributions

**Hend Mahmoud Abou El Nasr:** conceptualization, investigation, methodology, data curation, formal analysis, visualization, writing original draft, and writing – review and editing. The author conceived, conducted, analyzed, and reported the work described in this manuscript.

## Funding

No funding was received for this manuscript.

## Disclosure

The author has read and approved the final version of the manuscript. Corresponding author Hend Mahmoud Abou El Nasr had full access to all the data in this study and takes complete responsibility for the integrity of the data and the accuracy of the data analysis.

## Conflicts of Interest

The author declares no conflicts of interest.

## Supporting information


**Supporting Information 1** Additional supporting information can be found online in the Supporting Information section. File S1: CARE checklist of information to include when writing a case report.

## Data Availability

The data that support the findings of this study are available from the corresponding author upon reasonable request.
